# COVID-19 infection and oxidative stress: an under-explored approach for prevention and treatment?

**DOI:** 10.11604/pamj.2020.35.2.22877

**Published:** 2020-04-29

**Authors:** Marie-Pierrette Ntyonga-Pono

**Affiliations:** 1 d´Endocrinologie, Métabolisme, Faculté de Médecine de Libreville, Libreville, Gabon

**Keywords:** Oxidative stress, Covid-19, antioxidants, glutathione

## To the editors of Pan African Medical Journal

Oxidative stress is the result of an imbalance in the body between the oxidizing system, consisting mainly of free radicals, reactive oxygen species (ROS) and reactive nitrogen species (RNS) [1], and antioxidant systems that neutralize these free radicals capable of multiple deleterious effects. This oxidative stress is involved in aging [2] and is found in certain chronic pathologies such as diabetes mellitus, cancers, hypertension, coronary heart disease, etc. [3] and certain infections, particularly by the RNA viruses [4], a family to which belong corona viruses [5]. The objective of this work is to explain the role of oxidative stress in RNA virus infections and probably also in Covid 19 infection, in order to propose measures for prevention and treatment of this deadly infection which has already caused more than 118000 deaths worldwide [6]. This is an analysis of literature about oxidative stress, ways to counteract it, known links with certain RNA viruses and possible links with the new Corona virus.

**Oxidative stress and reactive oxygen and nitrogen species (RONS)**: reactive Oxygen and Nitrogen Species (RONS) are molecules characterized by the presence of unpaired valence electrons, which cause them to react with various biological molecules [7,8]. Main ROS are hydroxyl radical (OHº), superoxide anion (O_2_º-), singlet oxygen (¹O_2_), oxygen peroxide (H_2_O_2_) and ozone (O_3_), a powerful oxidant formed by endothermic reaction from O_2_ [8]. For RNS it is nitric oxide (NO) peroxynitrite (ONOO^-^), the nitrosyl cation (NO^+^), the nitrosyl anion (NO-), nitrous acid (NH_2_O_2_) .... [1,8] These free radicals are natural byproducts of various cellular processes and the functioning of structures such as mitochondria and the endoplasmic reticulum [4]. Under physiological conditions these reactive species play an important role in cell signalling, regulation of cytokines, growth factors, as immunomodulators, etc [1] and are involved in the natural aging of the human organism [2]. But when the balance is broken between oxidizing agents and antioxidant systems, which characterizes oxidative stress, these free radicals will have deleterious effects on all biomolecules [7,8]. The most reactive, hydroxyl radical can oxidize various molecules in its proximity, including DNA, phospholipids, and proteins. The superoxide can generate other free radicals and come into contact with nitric oxide (NO) to give the peroxynitrite radical (ONOO^-^), a powerful oxidant with NO depletion. Hydrogen peroxide is converted to hydroxyl and can cross cell membranes. Ozone is a powerful oxidant of lipid chains, it can generate other free radicals, and interact with a large number of organic and inorganic compounds [8]. Damage caused by these free radicals will affect cell membranes through the phenomenon of lipid peroxidation, oxidation and denaturation of proteins, DNA damage which can induce inflammatory immune responses, mutations and tumorigenesis risk, apoptosis [4]. So oxidative stress is involved in the occurrence of certain pathologies such as cancers, autoimmune diseases, cataract, Alzheimer’s and neurodegenerative diseases, diabetes mellitus, cardiovascular diseases, chronic kidney disease etc [2,3,8].

**What are the situations promoting oxidative stress?** about our subject, it is known that oxidative stress is triggered by a wide variety of viral infections [4,7] including HIV 1, viral hepatitis B,C,D viruses, herpes viruses, respiratory viruses, most of the RNA viruses [7] probably also corona viruses belonging to this family. Let us remind that corona viruses are encapsulated RNA viruses with different types: the classic coronaviruses, responsible for moderate respiratory infections in general, the SARS-CoV and MERS-CoV involved in epidemics of more severe respiratory infections [5] and the new coronavirus (SARS-CoV2) discovered in January 2020 responsible for infectious disease called COVID-19 which is currently experiencing a worldwide outbreak [6]. Generally, viral infections lead to an increase in production of free radicals and a depletion of antioxidants [1]. The mode of action varies according to the viruses as demonstrated by the analysis of the oxidative stress induced by different viruses of the flaviviridae family [4] but we find these two phenomena increasing the oxidative stress in these RNA viruses infections and for Ivanov [7] one of the sources of production of these ROS could be the mitochondrial dysfunction caused by the penetration of the virus into the cell. A “cytokine storm” with release of Il-2, Il-6, Il-7, TNF α etc. as been described in COVID-19 [9]. These authors described a cytokine shock with hyperinflammation accompanied by cytopenia, hyperferritinemia, [1,8] which is known to generate by the Fenton reaction (Fe²+ + H_2_O_2_→ Fe³+ + HO‾ + HO‾) the production of ROS [7,8]. In addition, cytokines and endotoxins will stimulate one of the isoforms of nitric oxide synthetase (NOs), the inducible isoform iNOs, which will stimulate the production of nitric oxide NO which will react with the superoxide ion to give the powerful oxidizing peroxynitrite radical (ONOO‾) [1,8]. Other factors can promote the endogenous production of free radicals, such as intense physical activity, high blood pressure, tissue ischemia, the action of certain metals (lead, arsenic, cesium, mercury) counteracting the co-factors of antioxidant enzymes, notably superoxide dismutase, NADPH oxidase, myeloperoxidase which will lead to the production of the superoxide radical. Physical agents (ionizing radiation, UV), solvents, various pollutants, an anesthetic agent halotane and even paracetamol have also been incriminated in this genesis of free radicals [8]. Several techniques can be used to evaluate the state of oxidative stress such as electron paramagnetic resonance, direct evaluation of oxidative stress markers such as oxidized glutathione, malonyl aldehyde, quantification of total antioxidant status etc [7].

**How are these free radicals neutralized?** there are multiple mechanisms to neutralize these free radicals: glutathione a natural antioxydant which has also an effect on viral replication [10], certain vitamins such as vitamin E and C, carotenoids and polyphenol with scavenging effect, [8] the glutathione peroxidase| glutathione reductase system allowing reduced glutathione (GSH) to bind to free radicals giving oxidized glutathione which will be regenerated into GSH through this system, super oxide dismutase (SOD) neutralizing superoxide anion (O_2_o‾), catalase eliminating H_2_O_2_, the peroxyredoxin system that neutralizes the peroxidation of lipids, protecting them from oxidative damage. Some trace elements such as Zinc and Selenium have an anti-oxidant effect as co-factors of anti-oxidant enzymes [1,7,8].

**What about covid-19 and oxidative stress?** SARS-CoV2, probably like other RNA viruses [4] can trigger an oxidative stress. This hypothesis can easily be checked by the dosage of oxidative stress markers [7] in the blood of sick people of COVID-19. A cytokin storm with hyper inflammation had been found in these patients [9] but researchers should also chek for a possible oxidative storm with all he deleterious effects of RONS, notably lipid peroxydation and proteins oxidation of membranes which can contribute to the transformation, hyalinization of pulmonary alveolar membranes [11] with letal respiratory distress. As elders and people suffering of diabetes, hypertension and cardiovascular diseases have already a state of oxidative stress [2,3], viral infection will increasae this stress, giving us one possible explanation of the severity of COVID-19 in these categories of patients [12].

**Suggestions for prevention and treatment of covid-19 infection:** in frail people, we propose to reduce their level of oxidative stress by providing them with substances that increase their antioxidant system [2] such as Glutathione, some trace elements like Zinc and Selenium, vitamin E and C, carotenoids and polyphenols [1]. Glutathione has analogues and precursors such as N acetyl cysteine. Indeed, cysteine is one of the three constituent amino acids of the major natural antioxidant Glutathione which also has an immunomodulating effect and destructive action on viruses such as herpes, influenza etc. by blocking viral replication [10]. There are also many antioxidants food, and even food additives such as butylated hydroxyanisole, quercetin and curcumin [1] could also be tested. In sick people, in addition to the various used treatments, we suggest to add antioxidants mentionned above and especially, injectable N acetyl cysteine [10] which has shown its effectiveness in hemorrhagic dengue fever [13] another RNA virus infection [4]. Various antioxidant which have been experimentally used successfully as melatonin, minocycline [1], can also be tested.

## Conclusion

In the fight against Covid-19 infection, all the possible treatments deserve to be taken into account. Relying on the complex pathophysiology of this viral infection, we suggest tu use also antioxydants agents in the treatment.

## Competing interests

The authors declare no competing interests.
